# ADP-Net: a hierarchical attention-diffusion-prediction framework for human trajectory prediction

**DOI:** 10.3389/frai.2025.1690704

**Published:** 2025-11-27

**Authors:** Zhenggui Zhang, Shanlin Xiao, Zhiyi Yu

**Affiliations:** School of Microelectronics Science and Technology, Sun Yat-sen University, Guangzhou, China

**Keywords:** representation learning, graph diffusion convolution, trajectory prediction, graph neural networks, spatio-temporal relational modeling, multi-hop, Personalized PageRank

## Abstract

Accurate prediction of human crowd behavior presents a significant challenge with critical implications for autonomous systems. The core difficulty lies in developing a comprehensive computational framework capable of effectively modeling the spatial-temporal dynamics through three essential components: feature extraction, attention propagation, and predictive modeling. Current spatial-temporal graph convolutional networks (STGCNs), which typically employ single-hop neighborhood message passing with optional self-attention mechanisms, exhibit three fundamental limitations: restricted receptive fields due to being confined to limited propagation steps, poor topological extensibility, and structural inconsistencies between network components that collectively lead to suboptimal performance. To address these challenges, we establish the theoretical connection between graph convolutional networks and personalized propagation neural architectures, thereby proposing attention diffusion-prediction network (ADP-Net). This novel framework integrates three key innovations: (1) Consistent graph convolution layers with immediate attention mechanisms; (2) Multi-scale attention diffusion layers implementing graph diffusion convolution (GDC); and (3) Adaptive temporal convolution modules handling multi-timescale variations. The architecture employs polynomial approximation for GCN operations and implements an approximate personalized propagation scheme for GDC, enabling efficient multi-hop interaction modeling while maintaining structural consistency across spatial and temporal domains. Comprehensive experiments on standardized benchmarks (ETH/UCY and Stanford Drone Dataset) show cutting-edge results, with enhancements of 4% for the average displacement error (ADE) and 26% for the final displacement error (FDE) metrics when contrasted with prior approaches. This advancement provides a robust theoretical framework and practical implementation for crowd behavior modeling in autonomous systems.

## 1 Introduction

Accurate modeling of pedestrian crowd dynamics underpins socially intelligent navigation systems (Yang et al., 2024; Pellegrini et al., 2009). Existing approaches capture local interactions adequately but lack the mechanisms to incorporate long-range social dependencies, leading to suboptimal predictions in dense pedestrian scenarios where distant influences significantly affect trajectory formation.

This limitation becomes apparent in human interactive navigation: two pedestrians walking toward each other may maintain separate paths until one accelerates, prompting anticipatory adjustments several meters before personal space is breached. This behavior demonstrates humans' innate ability to process non-local spatial dependencies, which current computational models cannot fully replicate for two reasons:

1. Existing methods have been constrained to local interactions, inherently limiting perceptual range.
2. Current architectures cannot effectively encode higher-order interactions (such as multi-hop influence propagation) into scalable embedding (Wu et al., 2023), nor achieve spectrally consistent adjacency-attention mappings. In simpler terms, most existing models struggle to look beyond immediate neighbors to capture more complex, multi-step relationships in a network, and they also find it hard to keep the learned attention patterns aligned with the network's true structural patterns.

Consequently, predictions lack anticipatory intelligence observed in real human navigation, where agents continuously integrate contextual signals across extended receptive fields.

Current trajectory prediction approaches typically employ recurrent neural networks (RNNs) for sequence modeling, social pooling for interaction capture, or graph message passing for relational reasoning (Defferrard et al., 2016; Kipf and Welling, 2017). While graph-based deep learning offer powerful representation learning capabilities, prevailing implementations face three fundamental constraints:

1. *Receptive field restriction*: most spatial-temporal GCNs confine message passing to direct neighbors (Gasteiger et al., 2019; Klicpera et al., 2019a), neglecting higher-order influences.
2. *Contextual scope limitation*: attention mechanisms compute weights solely over adjacent nodes (Wang et al., 2021), omitting critical non-local cues.
3. *Propagation degradation*: deeper architectures induce over-smoothing or over-squashing (Di Giovanni et al., 2023; ud din and Qureshi, 2024), impairing multi-hop reasoning.

A promising theoretical pathway emerges from the connection between GCNs and random walks. Standard GCNs with sufficient propagation steps converge to root invariant distributions (Xu et al., 2018). This property is detrimental for trajectory forecasting, where origin-awareness is essential. The personalized PageRank (PPR) framework (Page et al., 1999) resolves this issue through random walks with restart probability α ∈ (0, 1]. By probabilistically resetting walks to the root node (teleportation), PPR preserves locality (high α) while enabling multi-hop propagation (low α), formally establishing the foundation for our adaptive interaction mechanism.

However, operationalizing this theory faces significant challenges: (1) The inherent entanglement of propagation and feature extraction in message passing complicates multi-scale modeling; (2) Expanding neighborhood size amplifies computational complexity while risking information dilution; (3) Maintaining temporal consistency across spatial scales requires careful architectural design.

To overcome these limitations, we establish a unified framework through the asymptotic alignment of adjacency and attention matrices under graph diffusion principles, leveraging machine learning to design attention matrices whose spectral properties asymptotically align with those of diffused adjacency matrices via PageRank diffusion. This fundamental connection integrates: (1) GCN's capacity for localized convolution; (2) The strength of graph diffusion convolution (GDC) for multi-hop feature extraction, thereby enabling the simultaneous modeling of direct neighbor interactions and non-adjacent contexts via spectrally consistent propagation. A detailed explanation and proof are provided in Appendix A. We introduce the attention diffusion-prediction network (ADP-Net)—a novel framework that: (1) *Architecturally decouples* feature extraction from propagation through stage-wise processing; (2) *Theoretically unifies* graph attention, diffusion mechanisms, and personalized propagation; (3) *Hierarchically cascades* the integration of: (i) graph convolution with immediate attention for local interactions; (ii) multi-scale diffusion using graph diffusion convolution (GDC); (iii) adaptive temporal convolution for multi-timescale variations.

**Figure 1:**
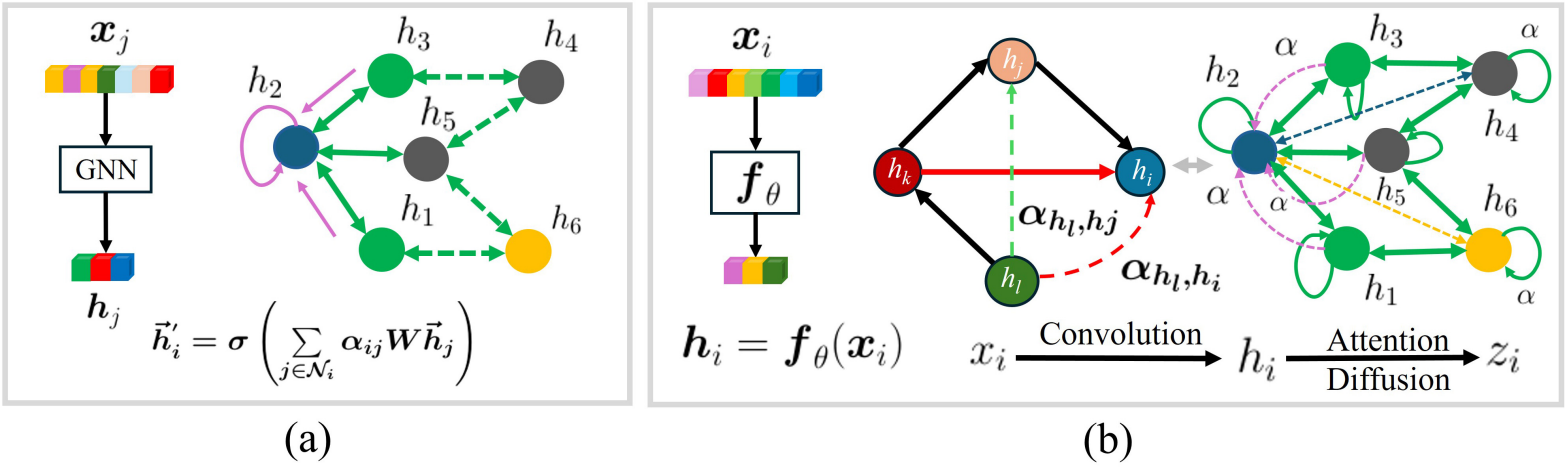
Revised the graph schematic to more accurately represent edge semanticsAdded clear visual indicators for node self-loop connectionsEnhanced the diagram layout for better interpretability of graph structure.

As shown in Figure 1, ADP-Net's hierarchical architecture enables attention to critical agents across extended receptive fields (Figure 1b), overcoming the single-hop constraint of conventional methods (Figure 1a). This approach effectively addresses the core challenge of *jointly modeling proximate interactions and distant contextual influences* without compromising spatial or temporal fidelity.

Rigorous evaluation and ablation studies demonstrate that ADP-Net achieves the improvements of 4% in mean average displacement error (ADE) and 26% in mean final displacement error (FDE) metrics, respectively, compared against existing methods while maintaining a favorable equilibrium of model complexity vs. performance.

This study makes three key contributions, which can be outlined as follows:

- This study introduces an innovative framework for trajectory forecasting, which synergistically combines graph convolutional networks, attention-based diffusion processes, and personalized neural prediction propagation. By leveraging machine learning to learn attention matrices whose spectral properties asymptotically align with diffused adjacency matrices via PageRank diffusion, we establish a unified framework combining GCN's local convolution with GDC's multi-hop extraction through spectrally consistent propagation. This innovation addresses three critical limitations in current graph convolution approaches: (1) restricted information propagation confined to immediate neighbors; (2) susceptibility to noise in real-world graph structures; and (3) sensitivity to arbitrarily defined neighborhood boundaries.
- Building upon this theoretical foundation, we develop attention-diffusion-prediction network (ADP-Net)—an architecturally optimized solution that sequentially combines graph convolution operations, attention diffusion processes, and predictive modeling. The network architecture achieves enhanced trajectory prediction accuracy through three key design innovations: (1) multi-scale neighborhood aggregation via graph diffusion; (2) adaptive attention weighting for dynamic relationship modeling; and (3) parameter-efficient prediction modules with personalized propagation schemes.
- Extensive experimentation on popular pedestrian benchmark datasets, namely ETH/UCY and Stanford drone dataset (SDD) have been conducted. Quantitative results and comparative analyses substantiate the competitive advantages of our method against leading contemporary approaches, particularly in forecasting accuracy and computational efficiency.

The study is structured as follows: Section 2 systematically reviews prior research in trajectory prediction with graph-based methods. In Section 3, we present our theoretical analysis of the graph diffusion-based multi-hop attention mechanism. The proposed framework and its technical innovations are detailed in Section 4. Section 5 provides comprehensive evaluations on benchmark datasets with in-depth analysis of the results. Finally, Section 6 concludes the study with key findings and future directions.

## 2 Related work

Trajectory prediction requires modeling complex spatio-temporal interactions from movement data, including: (1) agent-specific histories, (2) social group dynamics, (3) environmental constraints, and (4) scene semantics. Existing approaches often fail to coherently integrate these dimensions due to their inherent multi-scale nature.

Our work bridges this gap through a theoretical breakthrough, demonstrating that adjacency and attention matrices achieve asymptotic equivalence under graph diffusion. This equivalence implies that local neighbor information (captured by adjacency) and long-range dependencies (captured by attention diffusion) can be represented within a single spectral propagation operator. Consequently, multi-scale interactions—ranging from immediate agent collisions to scene-wide flow patterns—are modeled in a unified framework. This spectrally consistent propagation framework forms the basis for ADP-Net, which advances trajectory prediction by simultaneously processing two critical information pathways: (1) Direct neighbor interactions through self-attention mechanisms; (2) Non-adjacent contextual relationships via diffusion-based propagation.

For example, in a crowded intersection, self-attention captures the direct influence of a nearby pedestrian suddenly stopping, while diffusion propagation integrates the subtle yet coordinated motion of a group further away—such as a crowd moving toward a crosswalk—whose influence arrives indirectly through multiple intermediate agents. This joint processing yields responsiveness to immediate hazards and anticipation of large-scale flow changes, leading to more stable predictions.

Early work like social-STGCNN (Mohamed et al., 2020) captured spatial relations through weighted adjacency matrices and temporal dynamics via TXP-CNN. Subsequent studies enhanced direct graph convolutions with attention mechanisms (Gasteiger et al., 2019), while recent advances formalized multi-hop diffusion (Klicpera et al., 2019a; Wang et al., 2021) and multi-scale learning (Xhonneux et al., 2020) to better capture long-range dependencies.

Specifically, Graph Diffusion Convolution (GDC) (Klicpera et al., 2019a) introduced a spectral formulation based on personalized PageRank, allowing features to propagate over multiple hops through a fixed diffusion kernel. This effectively extends message passing beyond immediate neighbors while maintaining local smoothness. However, GDC remains a linear propagation scheme with static diffusion weights, limiting its adaptability to dynamic, context-dependent interactions in crowd motion. Nevertheless, many of these methods still rely primarily on predefined neighborhood structures, which can restrict their flexibility in modeling complex, evolving interaction patterns.

To address these limitations, subsequent research has branched into two complementary directions: (1) Attention-augmented graph convolutions (Velickovic et al., 2018) for capturing local relationships; (2) Spectral multi-hop propagation (Klicpera et al., 2019a) for modeling global contexts. In contrast, our proposed framework integrates diffusion into a *learnable attention operator*, where the diffusion kernel is adaptively modulated by attention weights. This allows nonlinear and context-aware spectral transformations that extend beyond GDC's fixed filtering scheme.

Furthermore, we establish a spectral-domain equivalence theorem showing that the asymptotic behavior of our adaptive diffusion-attention operator converges to that of GDC under diffusion propagation. This result provides, for the first time, a unified spectral interpretation linking fixed diffusion and adaptive attention. Therefore, our framework not only generalizes GDC conceptually but also extends it theoretically, unifying diffusion-based global smoothing with attention-driven local adaptability within a single spectral propagation paradigm.

While hierarchical spatio-temporal methods later refined these approaches, they remain limited to explicit connections—failing to emulate human cognition's ability to integrate indirect contextual cues. Our key theoretical breakthrough proves the asymptotic equivalence of adjacency and attention matrices under graph diffusion, unifying: (1) GCN's local operations (Kipf and Welling, 2017); (2) GDC's spectral propagation (Klicpera et al., 2019a); (3) Polynomial-filtered convergence (Gasteiger et al., 2019).

This yields three advances: (1) A spectrally consistent framework for direct and multi-hop interactions; (2) Motion-aware attention capturing distant agent dynamics; (3) Stable prediction through diffused contextual integration.

## 3 Multi-hop attention mechanism with graph diffusion

We aim to understand the multi-hop attention mechanism with graph diffusion by analyzing node influence scores. Following Xu et al. (2018), in a *k*-layer GCN, the influence score between nodes equals the expected value of a scaled, adjusted *k*-step random walk from the source node. This distribution converges to a stationary distribution **π**_lim_, obtained by solving $\pi_{\text{lim}}=\hat{\tilde{A}}\pi_{\text{lim}}$ (where $\hat{\tilde{A}}$ is defined subsequently). Notably, this result depends solely on the graph structure and is independent of the starting node.

To operationalize this theoretical foundation, our ADP-net model represents agent features and relationships as dynamic graphs. We define the graph structure as G=(V,E) containing *N* vertices in V and edges E⊆V×V. Every edge carries weight via ψ:E→ℝ, while nodes are characterized by feature matrices.

Specifically for pedestrian trajectory prediction, we represent the dynamic environment at time *t* by an evolving graph $G_{t}=\left(V_{t},E_{t}\right)$. Here, $V_{t}=\left\{v_{t}^{i}|i=1,\ldots ,N\right\}$ denotes the set of pedestrians, whose nodal features are given by their locations ${\text{p}}_{t}^{i}=\left(x_{t}^{i},y_{t}^{i}\right)$. The edge set $E_{t}=\left\{e_{t}^{ij}|i,j\in \left\{1,\ldots ,N\right\}\right\}$ defines interactions, where $e_{t}^{ij}=1$ indicates the presence of a connection. To quantify interaction strength, we assign weights $a_{t}^{ij}$ via kernel functions, forming the weighted adjacency matrix $A_{t}\in {\mathbb{R}}^{N\times N}$. Thus, $G_{t}$ is fully described by ***A***_*t*_, with ***Ã***_*t*_ = ***A***_*t*_ + ***I***_*N*_ denoting the self-loop-augmented adjacency matrix.

Building on this representation, a widely adopted message passing scheme employs GCNs (Kipf and Welling, 2017). For two layers:


$$\begin{array}{l} H_{\text{GCN}}=\text{softmax}\left({\hat{\tilde{A}}}_{t}\text{ReLU}\left({\hat{\tilde{A}}}_{t}X_{v_{t}}W_{0}\right)W_{1}\right), \end{array}$$ (1)

where ${\hat{\tilde{A}}}_{t}={\tilde{D}}^{-1/2}{\tilde{A}}_{t}{\tilde{D}}^{-1/2}$ is the symmetrically normalized adjacency matrix. However, when extending GCN to capture larger neighborhoods critical for crowd dynamics, we confront two challenges: (1) Oversmoothing (Alon and Yahav, 2021) from excessive averaging, diminishing local sensitivity; and (2) Parameter inefficiency when expanding receptive fields.

These limitations motivate adopting diffusion-based influence quantification. In graph representation learning, Personalized PageRank (PPR) (Klicpera et al., 2019a) measures influence *I*(*x, y*) as the (*x, y*)-th entry of


$$\Pi_{\text{ppr}}=\alpha {\left(I_{n}-\left(1-\alpha \right)\hat{\tilde{A}}\right)}^{-1},$$

capturing multi-hop dependencies via matrix inversion.

PPR is a special case of the graph diffusion framework, where the propagation matrix ***T*** is typically $\hat{\tilde{A}}$, ***T***^*k*^ encodes *k*-hop transitions, and θ_*k*_ controls their weights:


$$Z=\overset{\infty }{\underset{k=0}{\sum }}\theta_{k}T^{k}X.$$

Here, ***X*** represents the node feature matrix, containing the features of all nodes in the graph. Setting $\theta_{k}^{\text{PPR}}=\alpha {\left(1-\alpha \right)}^{k}$ recovers PPR, showing it as one diffusion kernel under a unified spectral filtering view.

Our spatiotemporal model leverages this by (1) using spatial-temporal convolutions for local motion encoding and (2) learning attention matrices whose spectra align with diffused adjacency matrices via PPR. This unifies GCN-style local aggregation and GDC-style multi-hop extraction: low-order attention captures immediate neighbors, while high-order terms follow diffusion probabilities. The resulting spectrally consistent propagation expands receptive fields dynamically—like adjusting a telescope—allowing distant yet relevant agents to influence predictions without losing local sensitivity.

## 4 Proposed framework

### 4.1 Problem formulation

Considering *N* pedestrians whose historical trajectories ${\left\{{\text{tr}}_{o}^{n}\right\}}_{n=1}^{N}$ over *T*_*o*_ timesteps, we predict future trajectories ${\text{tr}}_{p}^{n}={\left\{{\text{p}}_{t}^{n}\right\}}_{t=1}^{T_{\text{pred}}}$, where ${\text{p}}_{t}^{n}=\left(x_{t}^{n},y_{t}^{n}\right)$. To capture the inherent uncertainty in human motion, each predicted position is modeled as a bivariate Gaussian distribution:


$$\begin{array}{l} {\text{p}}_{t}^{n}\sim N\left(\mu_{t}^{n},\sigma_{t}^{n},\rho_{t}^{n}\right), \end{array}$$ (2)

with $\mu_{t}^{n}$ representing the mean value, $\sigma_{t}^{n}$ denoting the standard deviations, while $\rho_{t}^{n}$ indicates the correlation coefficient.

Based on this framework, our objective is to develop a trajectory prediction model $G_{\psi }\left(\cdot \right)$ that optimizes the probability of accurately forecasting future trajectories. This is accomplished through optimization of the negative logarithmic likelihood across all time steps and pedestrian instances:


$$\begin{array}{l} L\left(\psi \right)=-\overset{N}{\underset{n=1}{\sum }}\overset{T_{pred}}{\underset{t=1}{\sum }}\log\mathbb{P}\left({\text{p}}_{t}^{n}|{\hat{\mu }}_{t}^{n},{\hat{\sigma }}_{t}^{n},{\hat{\rho }}_{t}^{n}\right), \end{array}$$ (3)

where ψ denotes trainable parameters. The optimized model generates distributions that closely match ground truth trajectory statistics.

### 4.2 Framework introduction

**Figure 2:**
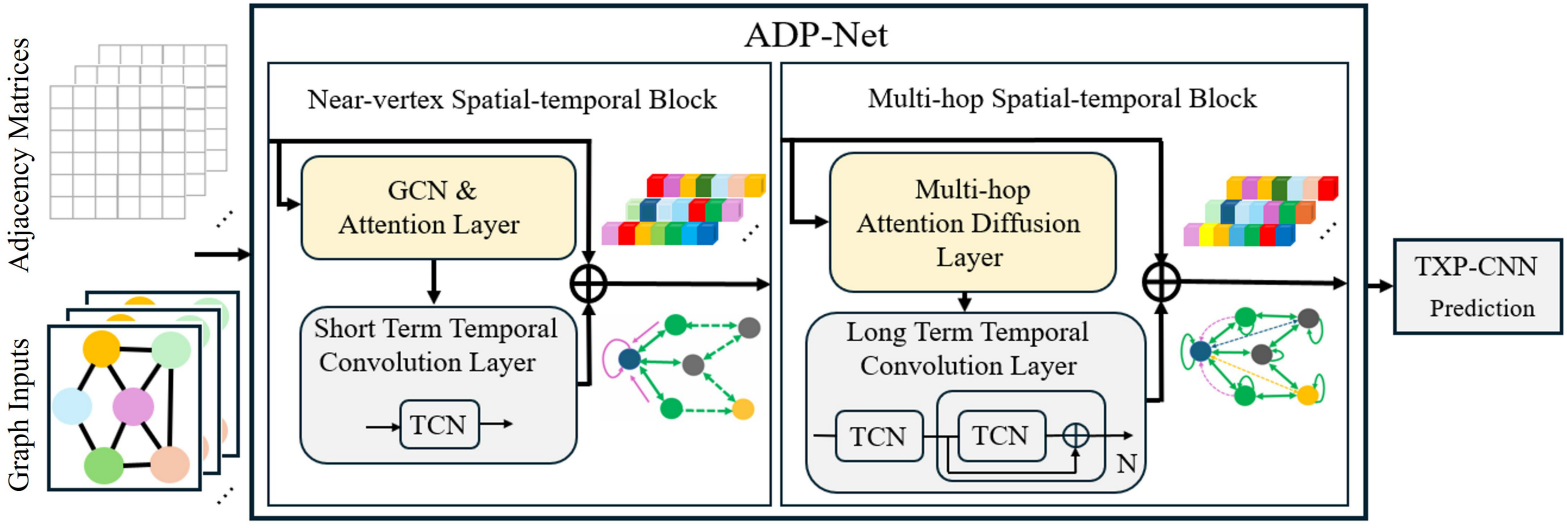
Updated to maintain consistency with the revised graph representation in Figure 1 Modified associated components to align with the improved visual conventions Ensured continuity in the graphical narrative across both figures.

The ADP-Net framework (Figure 2) employs two complementary spatio-temporal blocks for pedestrian trajectory modeling: (1) a Near-vertex Spatial-temporal Block, which combines Graph Convolutional Networks (GCNs) with temporal convolutions to model interactions among directly connected neighbors; and (2) a Multi-hop Spatial-temporal Block, which applies Graph Diffusion Convolution (GDC) with temporal convolutions to capture informative context from nodes that are not directly connected but influence the target through multi-hop structural relationships.

This design addresses critical limitations of existing approaches: While stacking GNN/GAT layers enlarges receptive fields, it induces over-smoothing/over-squashing issues (Wang et al., 2019; Oono and Suzuki, 2020). Instead, ADP-Net adopts GDC-based multi-hop propagation (Klicpera et al., 2019a) to efficiently incorporate non-adjacent context without deepening the network. The generalized graph diffusion in GDC acts as a polynomial filter (Gasteiger et al., 2019), suppressing noise in graph edges while capturing large-scale structural patterns. This synergistic integration of localized attention and multi-hop diffusion is evidenced by stable ADE/FDE metrics.

### 4.3 Near-neighbor attention via spectral graph convolution

The spatial-temporal feature extraction for immediate neighbors is implemented through spectral graph convolution. This section details the spatial convolution component, temporal convolution is addressed in Section 4.5.

#### 4.3.1 Spectral graph convolution formulation

Given the time-varying adjacency matrix $A_{t}\in {\mathbb{R}}^{N\times N}$, we define the normalized graph Laplacian:


$$\begin{array}{l} L_{t}=I_{N}-D^{-\frac{1}{2}}A_{t}D^{-\frac{1}{2}}, \end{array}$$ (4)

where $D_{ii}=\underset{j}{\sum }A_{tij}$ is the degree matrix. The spectral convolution on node features ***H*** ∈ ℝ^*N*×*f*^ follows:


$$\begin{array}{l} \Theta {*}_{G}H=U\Theta \left(\Lambda \right)U^{T}H, \end{array}$$ (5)

with ***U*** being eigenvectors of ***L***_*t*_, **Λ** the eigenvalue matrix, and Θ a spectral filter.

#### 4.3.2 Chebyshev polynomial approximation

Direct computation of Equation 5 is expensive. Following Defferrard et al. (2016), we approximate Θ(**Λ**) using *K*-order Chebyshev polynomials *T*_*k*_:


$$\begin{array}{l} \Theta \left(\Lambda \right)\approx \overset{K-1}{\underset{k=0}{\sum }}\theta_{k}T_{k}\left(\tilde{\Lambda }\right),\;\tilde{\Lambda }=\frac{2\Lambda }{\lambda_{\max}}-I_{N}, \end{array}$$ (6)

where λ_max_ is the largest eigenvalue of ***L***_*t*_. This yields:


$$\begin{array}{l} \Theta {*}_{G}H\approx \overset{K-1}{\underset{k=0}{\sum }}\theta_{k}T_{k}\left({\tilde{L}}_{t}\right)H,\;{\tilde{L}}_{t}=\frac{2L_{t}}{\lambda \max}-I_{N}. \end{array}$$ (7)

#### 4.3.3 Linear approximation for efficient attention

For efficient attention modeling, we adopt a first-order linear approximation (*K* = 1) of Equation 7 Kipf and Welling, (2017):


$$\begin{array}{l} \Theta {*}_{G}H\approx \theta_{0}H+\theta_{1}\left(L_{t}-I_{N}\right)H. \end{array}$$ (8)

Setting θ = θ_0_ = −θ_1_ and adding self-loops via $\tilde{A}t=A_{t}+I_{N}$, we derive the practical GCN formulation:


$$\begin{array}{l} \Theta {*}_{G}H=\theta \left({\tilde{D}}^{-\frac{1}{2}}\tilde{A}t{\tilde{D}}^{-\frac{1}{2}}\right)H, \end{array}$$ (9)

where ${\tilde{D}}_{ii}=\underset{j}{\sum }{\tilde{A}}_{tij}$.

#### 4.3.4 Attention layer implementation

The near-neighbor attention update at layer *l* is:


$$\begin{array}{l} H_{t}^{\left(l+1\right)}=\sigma \left(\underset{\text{attention aggregation}}{\underset{︸}{{\tilde{D}}^{-\frac{1}{2}}{\tilde{A}}_{t}{\tilde{D}}^{-\frac{1}{2}}H_{t}^{\left(l\right)}}}\Theta^{\left(l\right)}\right), \end{array}$$ (10)

where **Θ**^(*l*)^ ∈ ℝ^*f*^^(*l*)^×*f*^(*l*+1)^ represents learnable weights; $H_{t}^{\left(l\right)}\in {\mathbb{R}}^{N\times f^{\left(l\right)}}$ denotes input features (with $H_{t}^{\left(0\right)}=X$); σ(·) stands for nonlinear activation (e.g., ReLU). This implements local aggregation through the symmetric normalized adjacency, capturing immediate neighbor influences. Unlike the graph attention network (GAT), which learns feature-dependent attention coefficients via a separate attention function, our formulation employs a topology-guided static weighting derived from the normalized adjacency matrix ${\tilde{A}}_{t}$. This design ensures numerical stability and structural consistency with the diffusion-based propagation scheme introduced in Section 4.4, while still emphasizing local neighbor contributions in a learnable manner through **Θ**^(*l*)^.

### 4.4 Multi-hop attention via generalized graph diffusion

This section focuses on the spatial diffusion component for multi-hop neighbors, while temporal modeling will be addressed in Section 4.5.

#### 4.4.1 Generalized graph diffusion framework

To overcome limited receptive fields, we adopt generalized graph diffusion (Klicpera et al., 2019a; Gasteiger et al., 2019). The influence propagation between multi-hop nodes is formulated as:


$$\begin{array}{l} S=\overset{\infty }{\underset{k=0}{\sum }}\theta_{k}T^{k}\;\text{s.t. }\overset{\infty }{\underset{k=0}{\sum }}\theta_{k}=1,\;\theta_{k}>0. \end{array}$$ (11)

The convergence is guaranteed by a transition matrix ***T*** ∈ ℝ^*N*×*N*^, while the hopping decay is regulated by θ_*k*_. In the case of undirected graphs containing self-loops, we employ symmetric normalization to ensure stability during diffusion:


$$\begin{array}{l} T_{\text{sym}}={\left(w_{\text{ring}}I_{N}+D\right)}^{-1/2}(w_{\text{ring}}I_{N}+A_{t}){\left(D+w_{\text{ring}}I_{N}\right)}^{-1/2}, \end{array}$$ (12)

with *w*_ring_ > 0. This formulation subsumes PageRank (Page et al., 1999) and heat kernels as special cases.

#### 4.4.2 Attention diffusion mechanism

Building on the generalized diffusion framework in Equation 11, we design attention diffusion to capture multi-hop dependencies:


$$\begin{array}{l} A_{t}=\overset{\infty }{\underset{i=0}{\sum }}\theta_{i}T_{\text{sym},t}^{i}, \end{array}$$ (13)

where $\theta_{i}=\alpha {\left(1-\alpha \right)}^{i}$ (geometric decay) (Klicpera et al., 2019b). Here α ∈ (0, 1] is the teleport probability controlling the trade-off between locality and globality. The *i*-th power $T_{\text{sym}}^{i}$ encodes *i*-hop relational paths, systematically expanding the attention receptive field.

This formulation establishes asymptotic equivalence between attention and adjacency matrices under diffusion, creating a unified representation that inherently balances local neighbor influences with global crowd dynamics. The geometric decay coefficients induce a spectral low-pass filtering effect that amplifies coherent motion patterns while attenuating local noise.

The feature aggregation becomes:


$$\begin{array}{l} \text{AttDiff}\left(G,H_{t}^{\left(l\right)},\Theta \right)=A_{t}H_{t}^{\left(l\right)}, \end{array}$$ (14)

This differs from GCN's localized aggregation (Sec. 4.3) by integrating multi-scale context through diffusion, enabling dynamic receptive field adjustment based on interaction intensity.

For detailed theoretical analysis of the asymptotic equivalence, spectral-domain gain mechanism, and integration with spatiotemporal correlations, we refer readers to Appendix B.

#### 4.4.3 Multi-head diffusion architecture

**Figure 3:**
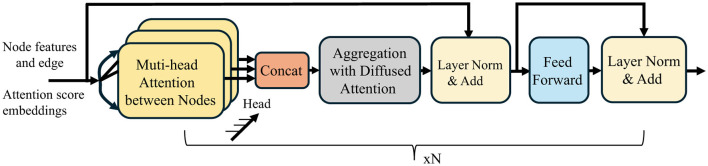
Architecture of the Multi-hop Attention Diffusion Layer, consisting of attention computation, diffusion-based multi-hop propagation, layer normalization, feed-forward layers, and dual residual connections.

Figure 3 presents a schematic diagram of the proposed Multi-head Diffusion architecture for multi-hop attention diffusion layer. To capture diverse relational subspaces, we extend Equation 14 to multi-head setting:


$$\begin{array}{l} \begin{array}{l} {\text{head}}_{i}=\text{AttDiff}\left(G,\text{LayerNorm}\left(H_{t}^{\left(l\right)}\right);\Theta_{i}\right),\\ \;{\hat{H}}_{t}^{\left(l\right)}=\text{Concat}\left({\text{head}}_{1},\ldots ,{\text{head}}_{M}\right)W_{O}, \end{array} \end{array}$$ (15)

where **Θ**_*i*_ parameterizes the *i*-th diffusion filter, and $W_{O}\in {\mathbb{R}}^{\left(M\cdot f\right)\times f}$ combines heads.

#### 4.4.4 Stable deep aggregation

Following Transformer design (Vaswani et al., 2017), we add layer normalization and residual connections:


$$\begin{array}{l} \begin{array}{l} {\hat{H}}_{t}^{\left(l+1\right)}=\text{LayerNorm}\left({\hat{H}}_{t}^{\left(l\right)}+H_{t}^{\left(l\right)}\right),\\ H_{t}^{\left(l+1\right)}=W_{2}\cdot \text{ReLU}\left(W_{1}{\hat{H}}_{t}^{\left(l+1\right)}+b_{1}\right)+b_{2}+{\hat{H}}_{t}^{\left(l+1\right)}, \end{array} \end{array}$$ (16)

where ***b***_1_, ***b***_2_ are bias vectors. This mitigates vanishing gradients while enabling deep feature fusion.

#### 4.4.5 Approximation for scalability

The exact computation of the diffusion operator $A_{t}$ has a complexity of $O\left(N^{2}\right)$ per evaluation. To improve scalability, we adopt an iterative approximation scheme following (Klicpera et al., 2019a):


$$\begin{array}{l} \begin{array}{l} Z_{t}^{\left(0\right)}=H_{t}^{\left(l\right)},\\ Z_{t}^{\left(k+1\right)}=\left(1-\alpha \right)T_{\text{sym},t}Z_{t}^{\left(k\right)}+\alpha Z_{t}^{\left(0\right)}\;\text{for }0\leq k<K-1,\\ Z_{t}^{\left(K\right)}=\text{softmax}\left(\left(1-\alpha \right)T_{\text{sym},t}Z_{t}^{\left(K-1\right)}+\alpha Z_{t}^{\left(0\right)}\right). \end{array} \end{array}$$ (17)

This procedure yields an approximation $Z_{t}^{\left(K\right)}\approx A_{t}H_{t}^{\left(l\right)}$, which converges to the true value as *K* → ∞ (Patel et al., 2020). The final softmax operation is applied to enhance the sparsity of the resulting attention weights, thereby improving model interpretability.

### 4.5 Multi-scale temporal convolution for pedestrian interactions

Building on the spatial features extracted in the previous section, we now shift our focus to modeling temporal dependencies through hierarchical temporal convolutions. Agent trajectories involve complex multi-scale temporal dependencies, ranging from quick, reactive movements that occur within sub-seconds to more strategic navigation behaviors spanning several seconds. Temporal Convolutional Networks (TCNs) provide an ideal framework for such tasks, with multi-layered architectures that utilize:

1. Dilated causal convolutions, which extend the temporal coverage while maintaining feature integrity by spacing kernels exponentially (*d* = 2^*l*^);
2. Strict causality enforcement, where future-masking via convolutional shifting ensures temporal coherence;
3. Hierarchical feature distillation, where successive layers transform immediate motions into higher-level navigational semantics.

#### 4.5.1 Short-term temporal modeling

**Figure 4:**
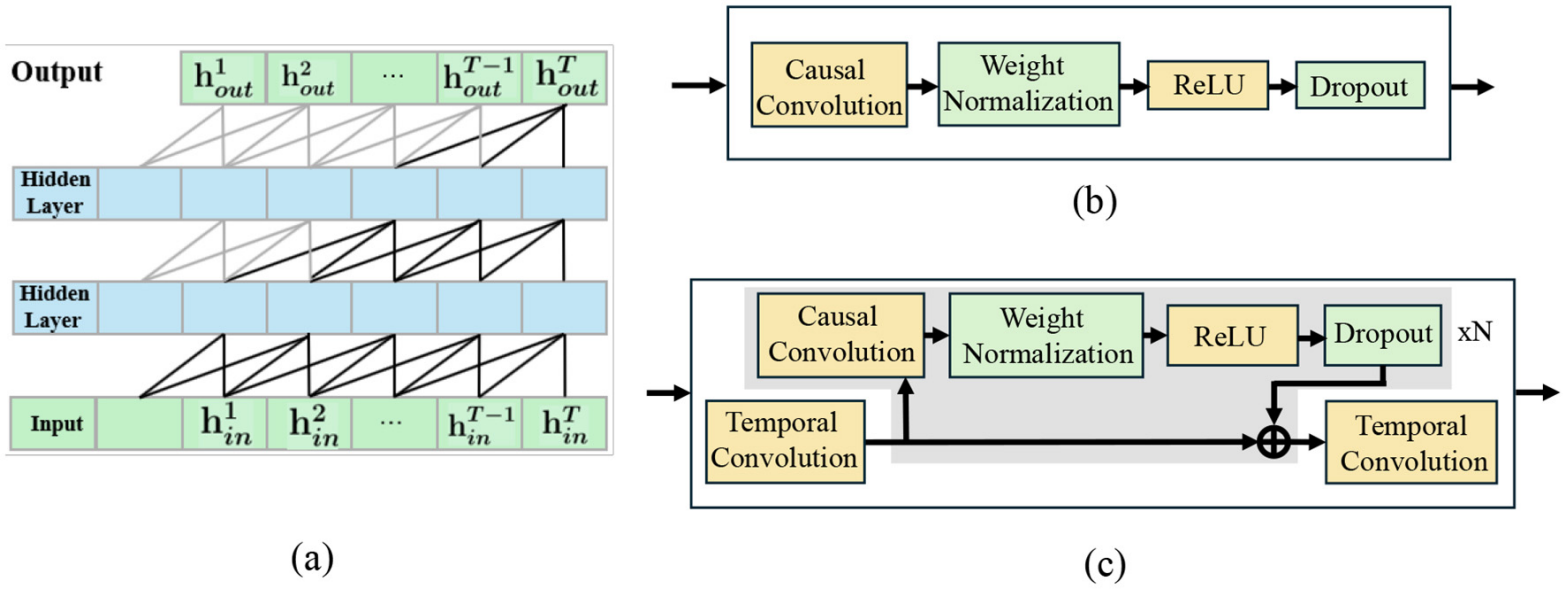
**(a)** Short-term TCN (three layers, kernel size = 3); **(b)** Residual refinement block integrating GCN output; **(c)** Long-term TCN (five layers, kernel size = 3) integrating GDC-enhanced features.

Based on the short-term TCN (three layers, kernel size = 3) (Figure 4a), for short-term temporal dependencies within the immediate node-attention aggregation window, we employ a lightweight Temporal Convolutional Network (TCN) module (Figure 4b) to model fine-grained motion dynamics. The short-term TCN module consists of three convolutional layers with kernel size = 3, stride = 1, and dilation rates of {1, 2}. This configuration yields a temporal receptive field of seven frames (approximately 2.8 s at 2.5 FPS), enabling the capture of immediate motion dynamics and short-term interaction patterns. This configuration enables precise temporal alignment and high-resolution feature extraction for short-term motion fluctuations without introducing redundant parameters. Despite the fixed dilation rate, the deeper hierarchy enables cumulative receptive-field expansion sufficient for the 12-frame observation horizon.

#### 4.5.2 Long-term temporal modeling

To capture comprehensive temporal dependencies across the 12-frame observation horizon, we implement a five-layer Temporal Convolutional Network with carefully calibrated parameters: kernel size = 3, stride = 1, and dilation rate = 1 across all layers. This configuration yields a receptive field of 11 frames, providing near-complete coverage of the 12-frame observation sequence while maintaining computational efficiency. The five-layer depth ensures sufficient feature abstraction capacity, while the consistent kernel size and dilation rate maintain temporal resolution integrity. This architecture effectively models both immediate motion dynamics and extended temporal patterns, enabling robust extraction of strategic navigation behaviors essential for accurate 12-frame trajectory prediction. Despite the fixed dilation rate, the deeper hierarchy enables cumulative receptive-field expansion sufficient for the 12-frame observation horizon. The residual refinement blocks further enhance these temporally enriched features, ensuring coherent propagation of interaction cues through the diffusion process, as illustrated in Figure 4c.

#### 4.5.3 Trajectory predictor

The refined features are passed into a novel CNN-based predictor (TXP-CNN), which extrapolates future trajectories. Given eight observed frames (≈3.2 s), the model predicts the next 12 frames, capturing the temporal continuity of pedestrian motion.

### 4.6 Architectural advantages of ADP-Net

#### 4.6.1 Non-local attention-diffusion mechanism

ADP-Net adopts a hierarchical Attention-Diffusion-Prediction framework, enabling trajectory forecasts based solely on historical observations. This framework extends the classical graph attention mechanism [e.g., GAT's ***H***^(*l*+1)^ = σ(***AH***^(*l*)^***W***^(*l*)^)]. Unlike GAT, which aggregates features strictly from direct neighbors via ***A***, ADP-Net asymptotically aligns adjacency and attention matrices through graph diffusion, thereby unifying local convolution with multi-hop feature extraction. The resulting A preserves parameter efficiency while replacing σ with layer normalization and deep aggregation, yielding higher expressive power than GAT's ELU-based aggregation.

#### 4.6.2 Spatial-spectral convolution integration

ADP-Net integrates spatial and spectral graph convolutions within a unified framework. It applies the normalized graph Laplacian ***L***_*t*_ for spectral convolution on $G_{t}$, and generalizes ***L***_*t*_ to the diffusion transformation matrix ${\tilde{T}}_{{\text{sym}}_{t}}$ via Graph Diffusion Convolution (GDC). This spectrally consistent propagation simultaneously models direct neighbor interactions and long-range, non-adjacent contexts. Although GDC is primarily spatial-based (Gasteiger et al., 2019), it admits a spectral interpretation, enabling ADP-Net to capture immediate spectral-spatial features before extracting multi-hop neighborhood information.

#### 4.6.3 Computational efficiency and stability

ADP-Net achieves high computational efficiency while maintaining stability. Unlike traditional spectral filtering methods, which incur a computational complexity of $O\left(N^{2}\right)$, ADP-Net leverages efficient approximation strategies to reduce computational cost. Following Kipf and Welling (2017), the core filtering operation exhibits a complexity of $O\left(|E|d^{\left(l\right)}d^{\left(l+1\right)}\right)$, where *d*^(*l*)^ and *d*^(*l*+1)^ denote the input and output feature dimensions, and |E| is the number of edges.

For attention-based diffusion implemented via GDC (Klicpera et al., 2019a; Wang et al., 2021), the complexity remains linear in the number of edges, i.e., O(|E|) (Wang et al., 2021). Moreover, the use of layer normalization in multi-head attention enhances training stability, which is further reinforced by the stability-oriented design of the temporal causal convolution module. Although the present analysis focuses on theoretical complexity, the linear dependence on |E| implies favorable scalability and practical efficiency for large-scale graphs.

#### 4.6.4 Modular extensibility for customization

ADP-Net is designed with modular extensibility in mind. Multi-hop diffusion blocks can be stacked with configurable skip connections, while standardized interfaces allow flexible insertion of BatchNorm, Dropout, and activation layers. Default components (e.g., the attention mechanism) can be replaced with novel modules without disrupting the overall architecture. This balance of flexibility and architectural consistency accelerates experimentation and facilitates adaptation to diverse trajectory prediction scenarios.

## 5 Experimental validation and findings

### 5.1 Experimental configuration

#### 5.1.1 Datasets

The performance evaluation of ADP-Net employs two benchmark datasets: the ETH/UCY collections and SDD (Stanford Drone Dataset).

- ETH/UCY: The ETH benchmark comprises two scenes (ETH and HOTEL), while UCY consists of three scenes (ZARA1, ZARA2, and UNIV). Pedestrian movements were recorded at 0.4-s intervals across an 8-s duration. During testing, the system utilizes an initial 3.2-s segment (eight frames) of visible motion paths to forecast the subsequent 4.8-s period (12 frames).
- SDD (Robicquet et al., 2016): a widely used benchmark, comprises multimodal trajectory data from diverse moving agents. This dataset records trajectories of over 11,000 pedestrian instances across twenty aerial-view scenarios. The annotations cover mixed traffic participants (pedestrians and vehicles) interacting in dense urban settings. All data was collected from naturalistic outdoor environments with complex social dynamics. Trajectories are sampled at 2.5 frames per second. For prediction tasks, The model needs to predict the next 12 positions based on 8 historical positions.

#### 5.1.2 Evaluation metrics

The experimental analysis employs a pair of quantitative metrics to evaluate the performance of our prediction model.

• Average Displacement Error (ADE) (Pellegrini et al., 2009): The mean spatial discrepancy measured by Euclidean distance across the entire forecast horizon, comparing predicted paths with their corresponding ground truth trajectories.


$$\begin{array}{l} \text{ADE}=\frac{\sum_{n\in N}\sum_{t\in T_{\text{pred}}}∥{\hat{p}}_{t}^{n}-p_{t}^{n}{∥}_{2}}{N\times T_{\text{pred}}} \end{array}$$ (18)

• Final Displacement Error (FDE) (Alahi et al., 2016) quantifies the spatial discrepancy by measuring the straight-line separation (Euclidean distance) of the predicted position from the ground truth when reaching the terminal prediction timestep.


$$\begin{array}{l} \text{FDE}=\frac{\sum_{n\in N}∥{\hat{p}}_{t}^{n}-p_{t}^{n}{∥}_{2}}{N},t=T_{\text{pred}} \end{array}$$ (19)

The ADE_*K*_ and FDE_*K*_ represent the minimum displacement error of *K* prediction results.

#### 5.1.3 Baseline comparison: generic and case-specific approaches

To demonstrate the effectiveness and advantages of ADP-Net, we first compare it with baseline methods in GNN representation learning, as well as with case-specific GNN variants that adapt graph structures for individual scenarios. We then provide a detailed discussion of the results.• S-GAN (Gupta et al., 2018) adopts a recurrent sequence-to-sequence architecture to process historical movement patterns and predict trajectories, featuring a novel pooling mechanism for aggregating multi-agent data.

• Sophie (Sadeghian et al., 2018) employs two complementary information sources: historical motion data encompassing both individual (Physical Attention) and interactive (Social Attention) behaviors of all agents in the scenario, along with environmental features extracted from images of the surroundings that provide scene context.

• PECNet (Mangalam et al., 2020), formally termed Predicted Endpoint Conditioned Network (PECNet), addresses human motion forecasting with enhanced adaptability. It estimates far-horizon path termination points to facilitate extended-range, probabilistic path prediction.

• Social-STGCNN (Mohamed et al., 2020) utilizes Social-STGCNN as an alternative to conventional feature aggregation methods through graph-based representation of interpersonal dynamics. The proposed framework improves trajectory forecasting performance, demonstrating superior results compared to existing approaches regarding accuracy, computational efficiency, and overall system simplicity.

• LB-EBM (Pang et al., 2021) utilizes an energy-guided probabilistic approach based on latent belief, where an objective function is formulated in the latent space to integrate both historical motion patterns and interpersonal dynamics, enabling the synthesis of multimodal trajectory forecasts.

• STNet (Wen et al., 2022) employs Graph Neural Networks (GNN), including Graph Attention Networks, combined with a transformer architecture employing a Conditional Variational Autoencoder (CVAE), to process social feature data derived from historical motion patterns and destination information in pedestrian trajectory forecasting.

• SKGACN (Lv and Yuan, 2023) utilizes a graph attention convolutional network guided by social knowledge (SKGACN) developed for modeling interpersonal dynamics and spatiotemporal dependencies between pedestrian trajectories, with optimized computational efficiency for prediction tasks.

• DTDNet (Liu S. et al., 2024) employs a hierarchical intention reasoning framework (Dynamic Target Driven Network, DTDNet) that processes pedestrian behaviors at varying timescales to model movement dynamics for trajectory forecasting.

• STS LSTM (Zhang et al., 2024) proposes a transferable STS-LSTM framework which captures pedestrian motion patterns by leveraging multi-domain features (spatial, temporal and spectral) for precise trajectory forecasting.

• Spatio-Temporal Adaptive Graph Pooling Network (STAGP) (Liu Z. et al., 2024) utilizes adaptive graph pooling for modeling dynamic interactions between individuals while pruning unnecessary edges. The framework further incorporates temporal feature extraction through spatio-temporal attention mechanisms, ultimately constructing STAGP (Spatio-Temporal Adaptive Graph Pooling Network) to forecast pedestrian movement patterns.

• V-Social STGCNN (Chang et al., 2024) considers the visual constraints of pedestrians during the construction of the weighted adjacency matrix, and proposes a novel trajectory forecasting method for pedestrians utilizing a visibility-aware spatiotemporal graph for prediction generation.

• SEI (Jiang et al., 2025) employs Social Entropy Informer for pedestrian trajectory prediction, which models both local and global interactions while using information entropy to capture the inherent randomness and uncertainty in human motion."

• RAN (Dong et al., 2025) proposes an iterative alignment mechanism employing a cyclic feature matching approach for comprehensive spatiotemporal synchronization of motion representations across instantaneous states and temporal evolution dimensions in trajectory forecasting.

• Two-Stage (Li and Zhang, 2025) employs a two-stage prediction method that combines multi-relation graph convolution, dynamic attention, and a global temporal aggregation module with LSTM and direction-change detection, capturing both local and global motion patterns while mitigating error accumulation.

• LG-STSCGN (Chen Y. et al., 2025) integrates a region-adaptive spatio-temporal graph with gate-controlled units in the time-sequence convolution module, establishing an extended-period gating framework for human motion forecasting using synchronized spatio-temporal graph networks.

• DSTIGCN (Chen W. et al., 2025) constructs a graph representation of spatial relationships and employs attention-based weighting to dynamically model pedestrian spatial correlations at every timestep, proposing a novel Deformable Spatial-Temporal Interaction Graph Convolution Network (DSTIGCN) to forecast future trajectories.

• FOV-aware (Zeng and Wang, 2025) employs a dynamic spatio-temporal graph along with a field-of-view (FoV-aware) masking mechanism that filters out irrelevant interactions by adaptively considering pedestrian distances and movement directions for prediction.

### 5.2 Implementation details

We developed our framework based on PyTorch (Paszke et al., 2019), with all experiments conducted on NVIDIA's V100 GPU. For optimization, we employed SGD (Stochastic Gradient Descent). Training proceeded for 250 epochs using a 128-sample batch size, where the learning rate began at 0.01 and reduced to 0.002 following epoch 150. Ablation results in Table 1 indicate that optimal performance is achieved with a single Multi-hop Spatio-temporal Block layer combined with two attention heads.

**Table 1:** The ablation study of GCN & attention only and Multi-hop Attention Diffusion Layers together on ETH/UCY dataset.

**Model**		**ETH**		**HOTEL**		**UNIV**		**ZARA1**		**ZARA2**		**MEAN**	
		**ADE**	**FDE**	**ADE**	**FDE**	**ADE**	**FDE**	**ADE**	**FDE**	**ADE**	**FDE**	**ADE**	**FDE**
Near-vertex Block		0.64	1.11	0.49	0.85	0.44	0.79	0.34	0.53	0.30	0.48	0.44	0.75
Multi-hop Block	LN = 1, HN = 1	0.50	0.57	0.30	0.47	0.13	0.14	0.30	0.57	0.22	0.30	0.30	0.35
	LN = 1, HN = 2	0.34	0.35	0.28	0.43	0.10	0.11	0.22	0.32	0.14	0.17	0.21	0.28
	LN = 2, HN = 1	0.41	0.57	0.28	0.43	0.11	0.12	0.36	0.54	0.19	0.25	0.27	0.38
	LN = 2, HN = 2	0.42	0.62	0.44	0.53	0.13	0.15	0.27	0.33	0.22	0.33	0.29	0.39

LN, layer number; HN, head number.

### 5.3 Ablation study

#### 5.3.1 Quantitative analysis of diffusion mechanism variants

We evaluate different architectural variants of the diffusion mechanism (varying layer numbers LN and attention heads HN) through quantitative metrics.

To assess the effectiveness of graph diffusion, we conduct ablation studies on the ETH/UCY datasets, comparing: (1) a baseline using a near-vertex spatial-temporal block layer, and (2) variants that incorporate multi-hop diffusion. As shown in Table 1, the optimal configuration combines one near-vertex spatial-temporal block layer with one multi-hop spatial-temporal block layer, utilizing two attention heads. The results indicate that incorporating multi-hop diffusion significantly improves the model's performance, particularly in capturing the interactions between agents that are connected in the graph, which may not always be spatially nearest but still provide critical network context. Compared to the baseline using only the nearest neighbor module (with average ADE/FDE of 0.44/0.75), incorporating a multi-hop diffusion module with dual-head attention (HN = 2) reduced the average ADE/FDE to 0.21/0.28, achieving relative improvements of 52% and 62%, respectively. This strongly validates the effectiveness of our proposed multi-hop attention diffusion mechanism. This improvement is most noticeable on the UNIV dataset, where agent density is higher than in other datasets, highlighting the method's effectiveness in densely connected environments.

#### 5.3.2 Qualitative comparison of module combinations

**Figure 5:**
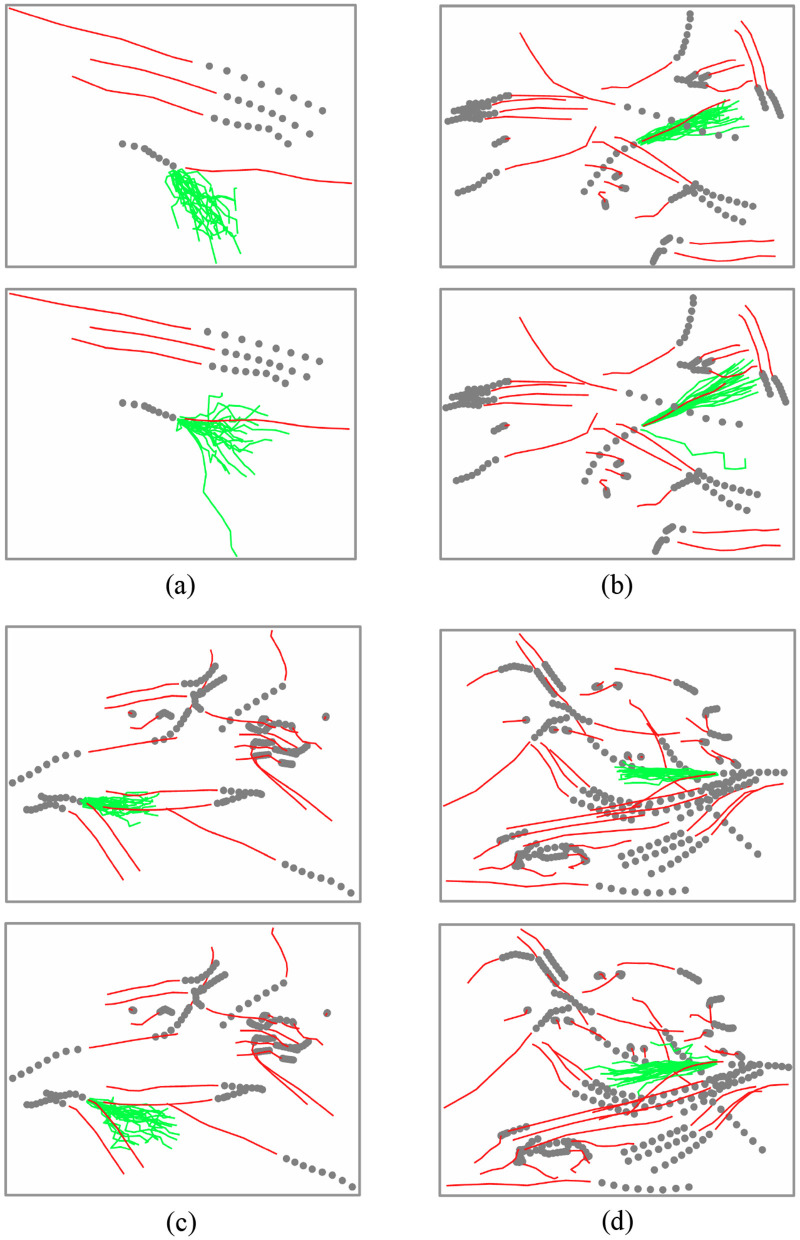
Comparison of trajectory predictions in four traffic scenarios: **(a)** bidirectional movement with 1-vs-3 counterflow, **(b)** collective right-turning with minimal deviation, **(c)** parallel runners making sharp turns to avoid oncoming pairs, and **(d)** complex multi-agent crowd dynamics. Dark gray dots show historical trajectories, red lines indicate ground truth, and 20 green lines denote predictions. For each scenario pair, the top row presents GCN-attention baseline results, while the bottom row shows GCN-attention with multi-hop diffusion.

We analyze the attention convolution module vs. its combination with attention diffusion. Figure 5 presents this comparison across four distinct scenarios. Each scenario includes: dim gray dots (historical trajectories), a solid red line (ground truth future trajectory), and 20 green lines (predicted trajectories). The top row of each panel displays results using only the GCN and attention block layer, while the bottom row shows results after adding the multi-hop attention diffusion layer.

**Figure 6:**
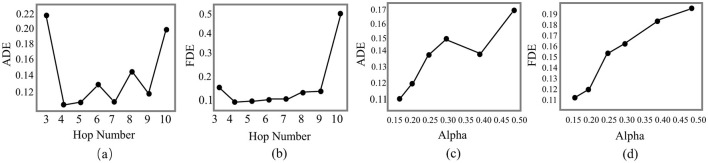
Analysis of ADP-Net on the UNIV dataset focusing on ADE and FDE performance: **(a, b)** effect of hop number; **(c, d)** effect of teleport probability α.

To maintain visual clarity, the figure omits complete predicted trajectories for all agents, focusing instead on a single agent of interest within each multi-agent scene. For non-focal agents, only their historical and ground truth future trajectories are shown. As evident in Figure 6, predictions incorporating the attention diffusion layer demonstrate closer alignment with the actual future trajectory, whereas predictions using only the GCN and attention layer exhibit significant deviations from the agent's true movement trend.

#### 5.3.3 Hyperparameter sensitivity analysis

Figure 6 presents the hyperparameter sensitivity analysis of hop number *k* and teleport probability α on model performance, with detailed results reported for the UNIV dataset. The analysis reveals three key findings:

(1) **Performance enhancement zone**—Performance improves notably when *k* increases from 4 to 5, provided that α ≤ 0.25.

(2) **Saturation effect**—Increasing *k* beyond 5 hops (up to 10) yields diminishing gains, indicating an optimal receptive field around *k* = 5.

(3) **Parameter sensitivity**—α exhibits a strict upper bound, with values above 0.25 causing performance degradation due to excessive localization. Based on these insights, we identify the following optimal hyperparameter settings:

(a) **Optimal hop number**: *k* = 5, balancing multi-hop context aggregation with computational efficiency.

(b) **Teleport probability range**: α ∈ (0.10, 0.25], ensuring an effective trade-off between local and global information.

These trends reflect the balance between information propagation depth and locality control. A moderate hop number (*k*≈5) expands the receptive field and enriches contextual information without introducing excessive noise, while an α in the range (0.10, 0.25] promotes long-range information flow while preserving essential local structures. Larger α values bias the model toward immediate neighbors, whereas smaller ones may introduce irrelevant distant nodes—both degrading performance. The slight oscillations observed as α increases arise from the local-global trade-off: greater local emphasis can help or harm depending on neighborhood quality, while excessive reliance on distant nodes introduces noise and instability. The heterogeneity of the graph amplifies these fluctuations, yet the overall trend remains a gradual performance decline with increasing α.

Unlike methods that rely on complex convolutional networks, ADP-Net tackles the key challenge of incorporating non-adjacent yet contextually relevant nodes—specifically those within the extended receptive field that influence the root node. Built on the same spatio-temporal GCN foundation as Social-STGCNN (7.6k parameters), our architecture enhances these techniques while maintaining comparable parameter efficiency (8.7k parameters). By relying solely on basic convolution operations, ADP-Net achieves superior computational efficiency (Mohamed et al., 2020).

To further ensure the robustness of ADP-Net under dynamic interaction scenarios, we reconstructed an interaction-level dataset covering various social behaviors (e.g., avoidance, diversion, and multi-directional intersection movements). Detailed configurations and extended results are provided in Appendix C.

### 5.4 Comparison with state-of-the-art methods

We quantitatively benchmark ADP-Net against leading approaches, including GNN-based, transformer-based, spectral-spatio-temporal, and other representative methods. The results on ETH/UCY (Table 2) show that ADP-Net outperforms the strongest GNN baseline (Two-Stage) by approximately 4% in ADE and 26% in FDE, achieving the best mean performance in both metrics.

**Table 2:** Min ADE_20_/min FDE_20_ for pedestrian trajectory forecasting (ETH-UCY benchmark).

**Model**	**GNN-based^α^ approaches**	**ETH**		**HOTEL**		**UNIV**		**ZARA1**		**ZARA2**		**MEAN**	
		**ADE**	**FDE**	**ADE**	**FDE**	**ADE**	**FDE**	**ADE**	**FDE**	**ADE**	**FDE**	**ADE**	**FDE**
Social-STGCNN (Mohamed et al., 2020)	GNN	0.64	1.11	0.49	0.85	0.44	0.79	0.34	0.53	0.30	0.48	0.44	0.75
STNet (Wen et al., 2022)	Transformer^β^	0.33	0.47	0.16	0.25	0.32	0.55	0.23	0.41	0.18	0.34	0.24	0.41
SKGACN (Lv and Yuan, 2023)	GNN	0.55	0.83	0.30	0.50	0.39	0.75	0.30	0.51	0.26	0.45	0.36	0.61
STS LSTM (Zhang et al., 2024)	Spectral^γ^	0.45	0.81	0.20	0.28	0.30	0.56	0.24	0.47	0.37	0.70	0.31	0.56
STAGP (Liu Z. et al., 2024)	GNN	0.65	1.21	0.41	0.73	0.38	0.68	0.28	0.46	0.25	0.44	0.40	0.70
V-Social-STGCNN (Chang et al., 2024)	GNN	0.61	0.95	0.30	0.44	0.37	0.64	0.32	0.52	0.30	0.49	0.38	0.61
SEI (Jiang et al., 2025)	Self-attention^δ^	0.34	0.64	0.19	0.33	0.29	0.61	0.24	0.52	0.22	0.46	0.26	0.51
RAN (Dong et al., 2025)	Pre-Aligned^ε^	0.41	0.69	**0.13**	**0.21**	0.25	0.46	0.22	0.41	0.16	0.31	0.23	0.42
LG-STSCGN (Chen Y. et al., 2025)	GNN	0.41	0.51	0.25	0.39	0.28	0.46	0.24	0.37	0.18	0.29	0.28	0.40
Two-Stage (Li and Zhang, 2025)	GNN	0.37	0.62	0.15	0.25	0.22	0.42	**0.21**	0.33	0.15	0.29	0.22	0.38
DSTIGCN (Chen W. et al., 2025)	GNN	0.43	0.70	0.22	0.41	0.25	0.45	0.20	0.37	0.17	0.32	0.25	0.45
FOV-aware (Zeng and Wang, 2025)	GNN	0.61	1.02	0.35	0.49	0.40	0.71	0.31	0.52	0.27	0.44	0.38	0.63
**Ours**		**0.34**	**0.35**	0.28	0.43	**0.10**	**0.11**	0.22	**0.32**	**0.14**	**0.17**	**0.21**	**0.28**

Bold values indicate superior performance.

^α^GNN-based methods (including all schemes marked ^β^–^ε^ for comparison).

^β^Social transformer comparison scheme.

^γ^Spatial-temporal-spectral feature comparison scheme.

^δ^Self-attention comparison scheme.

^ε^Domain-shift pre-alignment comparison scheme.

Key observations:

Robust local-nonlocal modeling: ADP-Net effectively captures both short-range and multi-hop dependencies through the synergy of adjacency-attention alignment and Graph Diffusion Convolution, leading to consistent reductions in ADE and FDE across all subsets. On the UNIV scene, ADP-Net achieves a 52% reduction in ADE over the strongest competitor, demonstrating its superior ability to handle complex multi-agent behaviors.

Narrowed ADE-FDE gap: ADP-Net achieves a significantly smaller ADE-FDE gap compared to the baselines, demonstrating greater stability across different datasets, especially for long-horizon forecasting.

Scene adaptability: ADP-Net surpasses strong GNN and transformer, and other baselines on ETH, UNIV, and ZARA2. This success stems from its cohesive and systematic design, in contrast to the scene-wise tailoring and fragmented modifications employed by others, demonstrating robust generalization to diverse spatial layouts and agent densities.

SDD results (Table 3) further validate ADP-Net's competitiveness. By generating 20 predictions over 12 future steps, it matches or outperforms models like DTDNet (Liu S. et al., 2024), PECNet (Mangalam et al., 2020), and the Latent Energy-Based Model (Pang et al., 2021). This success is attributed to the synergy between spectral interpretability and spatial feature extraction, enabling effective adaptation to diverse motion patterns and scene geometries.

**Table 3:** The minADE_20_/minFDE_20_ for trajectory forecasting (Stanford Drone Dataset).

	**Method**					
**Performance**	**S-GAN (Gupta et al., 2018)**	**Sophie (Sadeghian et al., 2018)**	**PECNet (Mangalam et al., 2020)**	**LB-EBM (Pang et al., 2021)**	**DTDNet (Liu S. et al., 2024)**	**Ours**
ADE_20_	27.23	16.27	9.96	9.03	9.2	**8.80**
FDE_20_	41.44	29.38	15.88	15.97	15.4	**13.64**

Optimal performance is indicated by boldface type.

## 6 Conclusion

In this work, we identify a fundamental limitation in graph-based trajectory prediction: existing methods struggle to reconcile multi-hop context capture with feature preservation. Through spectral analysis, we prove this stems from the spectral divergence between adjacency (local filtering) and attention (global diffusion) operators, which forces existing approaches to choose between over smoothing and restricted receptive fields.

First, we propose a unifying theoretical framework—by establishing the asymptotic equivalence between adjacency and attention matrices under diffusion—that enables spectrally consistent propagation. This framework integrates local filtering with global context aggregation by learning to align attention matrices with diffused adjacency matrices via PageRank diffusion. This learned alignment resolves the over smoothing-dilation trade-off without requiring heuristic graph sparsification.

Second, we design an implementable architecture (ADP-Net) that instantiates our framework through motion-conditioned attention diffusion, which weights neighbors via adaptive attention, and spectral filters that preserve node distinguishability across multi-hop propagation. Comprehensive experiments on standardized datasets (ETH/UCY, Stanford Drone Dataset) show state-of-the-art results, with improvements of 4% in ADE and 26% in FDE over existing methods.

Additional experiments on dynamic interaction scenarios (Appendix C) further validate ADP-Net's adaptability to complex, time-varying crowd interactions.

Looking forward, we will explore dynamic graph alignment for time-varying interactions and cross-modal diffusion to extend spectral consistency to heterogeneous agents such as vehicles and pedestrians.

In addition, future work will investigate robustness under partial occlusions and abnormal motion patterns, enabling ADP-Net to better handle challenging real-world scenarios with missing or irregular trajectory observations.

## Data Availability

Publicly available datasets were analyzed in this study. This data can be found at: ETH: https://data.vision.ee.ethz.ch/cvl/aess/; UCY: https://graphics.cs.ucy.ac.cy/research/downloads/crowd-data.zip; SDD: https://cvgl.stanford.edu/projects/uav_data/.
