# Supplementary material for: ADP-Net: a hierarchical attention-diffusion-prediction framework for human trajectory prediction
Source: Front Artif Intell. 2025 Nov 27;8:1690704. doi: 10.3389/frai.2025.1690704 (PMC12696591; doi:10.3389/frai.2025.1690704)
Supplement: Supplementary file 1 [file Data_Sheet_1.pdf]

ADP-Net: A Hierarchical Attention-Diffusion-Prediction Framework for Human Trajectory Prediction

Zhenggui Zhang [1], Shanlin Xiao [1,*] and Zhiyi Yu [1]

[1] *School of Microelectronics Science and Technology, Sun Yat-sen University, China.*

Correspondence*:
Shanlin Xiao
xiaoshlin@mail.sysu.edu.cn

A APPENDIX SPECTRAL ALIGNMENT

A.1 Setting

Let $G = (V, E)$ be an undirected graph with adjacency matrix $A \in \mathbb{R}^{n\times n}$. We construct the symmetric normalized transition matrix:

$$\tilde{A} = A + I, \quad \tilde{D} = \mathrm{diag}(\tilde{A}\mathbf{1}), \quad \hat{\tilde{A}} = \tilde{D}^{-1/2}\tilde{A}\tilde{D}^{-1/2}. \tag{A.1}$$

The matrix $\hat{\tilde{A}}$ is symmetric ($\hat{\tilde{A}} = \hat{\tilde{A}}^{\top}$) and satisfies $\|\hat{\tilde{A}}\|_2 \leq 1$; hence all eigenvalues $\lambda \in \sigma(\hat{\tilde{A}})$ lie in $[-1, 1]$.

A.2 Diffusion Kernels

The Personalized PageRank (PPR) diffusion kernel is

$$K_\alpha \;=\; \alpha \sum_{k=0}^{\infty} (1-\alpha)^k \hat{\tilde{A}}^k \;=\; \alpha\big(I - (1-\alpha)\hat{\tilde{A}}\big)^{-1}, \qquad \alpha \in (0, 1). \tag{A.2}$$

A.3 Learned Attention Parameterizations

$$W_\theta \;=\; \sum_{k=0}^{m} c_k(\theta)\,\hat{\tilde{A}}^k \;\triangleq\; q_{m,\theta}(\hat{\tilde{A}}), \tag{A.3}$$

where $c_k(\theta)$ are learnable coefficients. For multi-head attention,

$$W_\Theta \;=\; \sum_{h=1}^{H} \pi_h\, q_{m,\theta_h}(\hat{\tilde{A}}), \qquad \pi_h \geq 0, \quad \sum_{h=1}^{H} \pi_h = 1. \tag{A.4}$$

THEOREM A.1 (**Learned Spectral Alignment**). *Let* $\hat{\tilde{A}}$ *and* K_α *be as in* (A.1) *and* (A.2)*, with* $\sigma(\hat{\tilde{A}}) \subseteq [-1, 1]$. *For a learned weight matrix* W*:*

1. ***Eigenvector Alignment.*** *If $W = q(\hat{\hat{A}})$ for some polynomial q, then W and K_α share eigenvectors with $\hat{\hat{A}}$.*
2. ***Error Bound.*** *Let $c_k^\star = \alpha(1-\alpha)^k$ and $q_m^\star(\lambda) = \sum_{k=0}^m c_k^\star \lambda^k$. For any coefficients $\{c_k\}_{k=0}^m$,*

$$\|K_\alpha - W\|_2 \;\le\; (1-\alpha)^{m+1} \;+\; \max_{\lambda\in\sigma(\hat{\hat{A}})} \Big|\sum_{k=0}^{m} (c_k^\star - c_k)\,\lambda^k\Big|. \tag{A.5}$$

Moreover, since $|\lambda| \le 1$,

$$\|K_\alpha - W\|_2 \;\le\; (1-\alpha)^{m+1} \;+\; \sum_{k=0}^{m} |c_k^\star - c_k|. \tag{A.6}$$

3. ***Asymptotic Alignment.*** *If $\sum_{k=0}^m |c_k^\star - c_k| \to 0$ as $m \to \infty$, then $W \to K_\alpha$ uniformly on $\sigma(\hat{\hat{A}})$.*

PROOF. Let $\hat{\hat{A}} = \sum_i \lambda_i u_i u_i^\top$ be an eigen-decomposition with $\lambda_i \in [-1, 1]$.

(1) Eigenvector Alignment. For any polynomial q, spectral calculus gives $q(\hat{\hat{A}}) = \sum_i q(\lambda_i)\, u_i u_i^\top$, so $q(\hat{\hat{A}})$ shares eigenvectors with $\hat{\hat{A}}$. Moreover,

$$K_\alpha \;=\; \alpha(I - (1-\alpha)\hat{\hat{A}})^{-1} \;=\; \sum_i \frac{\alpha}{1-(1-\alpha)\lambda_i}\, u_i u_i^\top \;=\; f(\hat{\hat{A}}),$$

with $f(\lambda) = \frac{\alpha}{1-(1-\alpha)\lambda}$, hence K_α also shares the eigenvectors $\{u_i\}$.

(2) Error Bound. Write $W = q(\hat{\hat{A}})$ where $q(\lambda) = \sum_{k=0}^m c_k \lambda^k$. Add and subtract $q_m^\star(\hat{\hat{A}})$:

$$\|K_\alpha - W\|_2 \;\le\; \underbrace{\|K_\alpha - q_m^\star(\hat{\hat{A}})\|_2}_{\text{diffusion tail}} \;+\; \underbrace{\|q_m^\star(\hat{\hat{A}}) - q(\hat{\hat{A}})\|_2}_{\text{coefficient mismatch}}.$$

On the spectrum of $\hat{\hat{A}}$,

$$\|K_\alpha - q_m^\star(\hat{\hat{A}})\|_2 \;=\; \max_{\lambda\in\sigma(\hat{\hat{A}})} \alpha\Big|\sum_{k=m+1}^{\infty} (1-\alpha)^k\lambda^k\Big| \;\le\; \alpha \sum_{k=m+1}^{\infty} (1-\alpha)^k \;=\; (1-\alpha)^{m+1},$$

since $|\lambda| \le 1$. Similarly,

$$\|q_m^\star(\hat{\hat{A}}) - q(\hat{\hat{A}})\|_2 \;=\; \max_{\lambda\in\sigma(\hat{\hat{A}})} \Big|\sum_{k=0}^{m} (c_k^\star - c_k)\lambda^k\Big| \;\le\; \sum_{k=0}^{m} |c_k^\star - c_k|.$$

Combining the two displays yields (A.5), and the last inequality gives (A.6).

(3) Asymptotic Alignment. As $m \to \infty$, the tail term $(1-\alpha)^{m+1} \to 0$. If, in addition, $\sum_{k=0}^m |c_k^\star - c_k| \to 0$, then (A.6) implies $\|K_\alpha - W\|_2 \to 0$ uniformly over $\sigma(\hat{\hat{A}})$.

Multi-head Remark. For $W = \sum_{h=1}^{H} \pi_h q_{m,\theta_h}(\hat{\tilde{A}})$, define averaged coefficients $\bar{c}_k = \sum_{h=1}^{H} \pi_h c_k(\theta_h)$ and note $W = \sum_{k=0}^{m} \bar{c}_k \hat{\tilde{A}}^k$. Applying the above argument with $\{\bar{c}_k\}$ yields the same conclusions (1)–(3).

B APPENDIX THEORETICAL FOUNDATIONS AND MECHANISMS OF ATTENTION DIFFUSION

This appendix provides the detailed theoretical foundations underlying our attention diffusion mechanism, addressing the asymptotic equivalence, spectral-domain gain mechanism, and integration with spatiotemporal correlations.

B.1 Asymptotic Equivalence and Local-Global Balance

The asymptotic equivalence between attention and adjacency matrices under diffusion establishes a unified representation that inherently balances local and global interactions through three key mechanisms:

- **Local interactions** are captured by low-order terms ($i = 0, 1$) in the diffusion series $\boldsymbol{\mathcal{A}}_t = \sum_{i=0}^{\infty} \theta_i \boldsymbol{T}^i_{\text{sym},t}$, representing immediate neighbor influences essential for collision avoidance and short-range social interactions.
- **Global interactions** emerge through higher-order terms ($i \geq 2$), where $\boldsymbol{T}^i_{\text{sym}}$ encodes i-hop relational paths that capture crowd-level motion patterns, destination-oriented behavior, and scene-wide flow dynamics.
- **Adaptive balancing** is intrinsically controlled by the geometric decay $\theta_i = \alpha(1-\alpha)^i$:
  - When $\alpha \to 1$, the series emphasizes local interactions (low-order terms dominate)
  - When $\alpha \to 0$, the series incorporates more global context (higher-order terms contribute significantly)
  - The learned α parameter automatically discovers the optimal balance for pedestrian trajectory prediction in different scenarios

This balance is particularly crucial for pedestrian trajectories because agents must simultaneously: (1) react locally to immediate neighbors for collision avoidance, and (2) align globally with crowd flow patterns and destination constraints. In practice, this local–global balance is realized through a Graph Convolutional Network (GCN) equipped with Graph Diffusion Convolution (GDC), where the GCN captures structural interactions and the GDC component modulates the diffusion-based propagation strength.

B.2 Spectral-Domain Gain Mechanism

The geometric decay coefficients $\theta_i = \alpha(1-\alpha)^i$ create an exponential low-pass filter in the spectral domain. Representing the graph by its normalized Laplacian $\boldsymbol{L} = \boldsymbol{I} - \boldsymbol{T}_{\text{sym}}$, the diffusion process corresponds to a spectral filter:

$$g(\lambda) = \frac{\alpha}{\alpha + \lambda(1-\alpha)}, \tag{B.1}$$

where λ denotes Laplacian eigenvalues. This filter exhibits the following properties:

- **Amplifies low-frequency components** (small λ) associated with globally coherent motion patterns—these represent consistent crowd movements and destination-oriented behaviors that are highly predictive of future trajectories.
- **Attenuates high-frequency components** (large λ) corresponding to local noise, individual deviations, and momentary fluctuations—these typically represent stochastic variations with lower predictive value.

- **Provides adaptive feature selection** by automatically emphasizing motion components that are spatially consistent and temporally persistent, which are precisely the patterns most relevant for trajectory forecasting.

The gain mechanism $g(\lambda)$ thus serves as a theoretically grounded feature weighting scheme that enhances prediction stability and accuracy.

B.3 Integration with Spatiotemporal Correlations

Our diffusion formulation naturally couples spatial and temporal correlations, which is essential for modeling pedestrian motion:

Spatial Correlation Encoding:

- The graph structure through $\boldsymbol{T}_{\mathrm{sym}}$ inherently captures spatial relationships between pedestrians
- The Laplacian eigenvectors represent the natural vibration modes of pedestrian spatial configurations
- Low-frequency eigenvectors correspond to smooth, globally coherent spatial patterns

Temporal Correlation Incorporation:

- The time evolution of attention patterns $\boldsymbol{\mathcal{A}}_t$ across trajectory sequences ensures temporal persistence
- Motion intentions and social interactions evolve smoothly over time through the diffusion process
- The temporal dimension is integrated through sequential processing of graph states

Unified Spatiotemporal Representation: The diffusion process $\boldsymbol{\mathcal{A}}_t$ naturally couples spatial and temporal aspects—the spatial propagation through $\boldsymbol{T}^i_{\mathrm{sym}}$ operates on temporally evolving attention patterns, creating a coherent spatiotemporal representation that captures both immediate reactions and persistent motion intentions.

This theoretical foundation explains why our diffusion-based approach is particularly effective for pedestrian trajectory prediction, as it aligns with the fundamental characteristics of human motion behavior.

C APPENDIX INTERACTION-LEVEL DATASET RECONSTRUCTION AND SCENE-WISE ANALYSIS

This section provides additional details and analyses to address the evaluation of dynamic interactive scenarios. Building upon the preceding ablation and sensitivity studies, which focused on the architectural and hyperparameter robustness of ADP-Net, we further examine the model's capability to handle dynamic social interactions across diverse crowd scenarios. To this end, we reconstruct the ETH/UCY benchmark datasets according to **interaction intensity**, enabling a more fine-grained evaluation of prediction stability under varying levels of agent interaction. This extended analysis complements the previous experiments and provides additional insight into how interaction complexity affects the model's behavioral generalization.

Based on the intrinsic characteristics of the ETH/UCY datasets—specifically, **UNIV** featuring dense crowds and frequent interactions, **ZARA1** exhibiting moderate density with numerous turns, and **ZARA2** showing moderate density with substantial cross-walking patterns—we reconstructed each dataset into **low**, **medium**, and **high** interaction levels. The classification was determined by an interaction score incorporating both pairwise distance and relative velocity, with thresholds defined according to the corresponding quantile levels. The statistical properties of the reconstructed datasets are summarized in Table C.1.

Table C.1. Scene-level Interaction Statistics of Reconstructed ETH/UCY Datasets

Scene	Interaction Level	Number of Agents	Average NN Distance	Interaction Events	Interaction Score	Number of Segments	Duration (seconds)
UNIV	Low	26.52	1.19	72.6	59.65	177	204.0
	Medium	37.72	0.97	146.9	112.57	177	208.0
	High	46.66	0.86	236.8	172.76	182	110.0
ZARA1	Low	3.93	2.61	1.5	2.75	296	305.2
	Medium	6.05	1.53	9.5	9.10	296	341.2
	High	9.44	1.24	27.1	21.67	305	360.4
ZARA2	Low	6.16	2.58	14.0	10.75	346	420.4
	Medium	10.73	1.17	36.6	27.92	345	392.8
	High	13.69	0.96	64.2	46.70	356	363.6

Note: Average NN Distance represents the mean Euclidean distance to each agent's nearest neighbor across all frames, reflecting local crowd density. Interaction Events count the number of temporal instances where two agents come within a predefined proximity threshold, capturing encounter frequency. Interaction Score is a composite metric integrating spatial proximity and event frequency over time, quantifying overall scene interaction intensity. Interaction levels (Low, Medium, High) are derived from percentile thresholds of these scores within each scene.

Table C.2 presents the ADE/FDE results under varying levels of interaction intensity. The results indicate that ADP-Net maintains stable predictive accuracy across all interaction scenarios. Notably, the model achieves slight performance gains under low-interaction conditions compared to the unified-scene baseline, while exhibiting only moderate degradation as interaction intensity increases to medium and high levels. This overall trend suggests that the model generalizes smoothly across different behavioral densities without overfitting to specific crowd patterns.

The improvement observed in low-interaction settings arises from smoother pedestrian trajectories and more consistent neighbor relations. Under such conditions, the coupled graph convolution–diffusion layers propagate motion information spectrally consistently—preserving local geometry while diffusing attention

over stable global structures. This allows ADP-Net to emphasize relevant agents and suppress spurious correlations, yielding more coherent trajectory representations and higher prediction precision.

As interaction intensity increases, the graph topology becomes more volatile, with frequent trajectory crossings and rapid neighbor exchanges. Such fluctuations challenge the attention–diffusion mechanism, which must continuously adapt its relational weighting. Although the model dynamically rebalances these connections, the instability of the underlying interaction graph inevitably introduces uncertainty that no deterministic predictor can fully eliminate. Nevertheless, the observed performance variation remains limited ($\leq$ 0.05–0.08 m in ADE/FDE, corresponding to improvements up to 8% and degradations bounded within 20%), indicating that the performance loss is minor and physically reasonable given the level of interaction-induced uncertainty.

Further analysis reveals several scene-specific trends. Within the **UNIV** dataset, the medium-interaction subset performs slightly worse than the high-interaction subset. Although higher interaction often implies greater complexity, the structured flows in UNIV promote more predictable patterns that the model can exploit effectively. In contrast, the medium-interaction scenes contain irregular and intermittent trajectories that increase motion uncertainty. A similar observation holds for **ZARA1** and **ZARA2**: the open, unconstrained spatial layout of ZARA1 yields more abrupt direction changes, whereas ZARA2's corridor-like configuration supports smoother, more consistent motions.

It should be noted that these interaction-level analyses were performed without retraining specific to the reconstructed subsets. Therefore, the values primarily reflect relative trends rather than absolute performance metrics. Overall, the consistent pattern of moderate improvement and bounded degradation across different interaction intensities reflects the inherent behavior of the graph convolution–diffusion coupling—robust under stable connectivity, yet sensitive to rapid topological fluctuations.

Table C.2. Performance Comparison across Interaction Levels

**Dataset**	**Interaction Intensity**											
	**Low**				**Medium**				**High**			
	**ADE**	$\Delta\%\uparrow$	**FDE**	$\Delta\%\uparrow$	**ADE**	$\Delta\%\downarrow$	**FDE**	$\Delta\%\downarrow$	**ADE**	$\Delta\%\downarrow$	**FDE**	$\Delta\%\downarrow$
UNIV	0.097	3	0.106	3	0.112	12.1	0.126	15.3	0.111	10.5	0.126	14.6
ZARA1	0.202	8	0.304	5	0.247	12.4	0.371	16.2	0.254	15.3	0.383	19.7
ZARA2	0.133	5	0.163	4	0.155	11.3	0.196	15.6	0.159	13.8	0.201	17.7

Note: $\Delta\%$ denotes the performance change relative to the unified-scene baseline. An upward arrow ($\uparrow$) indicates improvement, while a downward arrow ($\downarrow$) indicates deterioration. ADE: Average Displacement Error, FDE: Final Displacement Error. The **unified-scene baseline** refers to the model's performance on the original dataset without interaction-intensity segmentation (see Table 2 in the main text).
